# Influence of Selected Factors on the Adsorption Layer Structure of Polyamino Acids and Their Block Copolymers at the Solid–Aqueous Solution Interface

**DOI:** 10.3390/molecules28248080

**Published:** 2023-12-14

**Authors:** Iwona Ostolska, Małgorzata Wiśniewska

**Affiliations:** Department of Radiochemistry and Environmental Chemistry, Institute of Chemical Sciences, Faculty of Chemistry, Maria Curie-Skłodowska University in Lublin, Maria Curie-Skłodowska Sq. 3, 20-031 Lublin, Poland

**Keywords:** polymer adsorption, polyamino acid, chromium(III) oxide, poly(aspartic acid), block copolymer, solid–liquid interface

## Abstract

The adsorption mechanism of different polymers containing ionic polyamino acids monomers in the chain structure at the solid–liquid interface was investigated. Initially, the influence of molecular weight and solution pH on simple polyamino acids (poly(L-aspartic acid) and poly(L-lysine) binding was determined. Considering the obtained dependencies, the polymer adsorption layer conformation was proposed in the systems containing block copolymers (both diblock and symmetrical triblock) consisting of polypeptide as well as poly(ethylene glycol) fragments. The presented studies focused on the application of two experimental methods. The polymer adsorption was carried out using the batch method and the adsorbate concentration was determined spectrophotometrically. Then, the turbidimetric measurements were taken. The analysis of the obtained results showed that the adsorption process of block copolymers depends on two factors. Firstly, the solution pH determines both the nature of the interactions of the copolymer structural units with the solid surface and the conformation of the polypeptide chains. The second parameter influencing the adsorption layer structure is the ratio of the lengths of both blocks. Introducing a short PEG fragment into the polymer main chain may improve the polymer adsorption properties by increasing the number of interactions with the adsorbent surface.

## 1. Introduction

Adsorption of macromolecular compounds on a solid surface is a very complex phenomenon, depending on many factors. To determine the mechanism of macromolecules binding at the solid–solution interface, the type and structure of the polymer chain, the surface properties of the adsorbent and the type of interactions occurring between the above-mentioned components should be analyzed. The most important parameters affecting the polymer film structure include the polymer molecular weight, the type and ionic nature of its functional groups, and the number and distribution of active centers on the adsorbent surface. Equally important is the proper selection of process conditions, such as pH and ionic strength of the solution, temperature and the presence of other substances, such as heavy metal ions or surfactant molecules [1,2].

The practical aspect of high-molecular compound adsorption on the solid’s surface is related to its application in the processes of stabilization or flocculation of colloidal systems. Obtaining durable solid suspensions is of key importance in the production of, among others, medicines, cosmetics, paints, printer inks and paper. This process may occur as a result of adding substances such as simple inorganic ions, surfactants, or macromolecular compounds. Then, their binding on the solid surface contributes to an increase in the repulsive forces between the particles. In the macromolecular compounds case, the suspension stabilization mechanism is closely related to the conformation of polymer chains adsorbed at the interface. The introduction of numerous macromolecules with charged functional groups stretched towards the bulk phase onto the adsorbent surface (electrosteric stabilization) or the creation of a densely packed adsorption layer made of a non-ionic polymer (steric stabilization) leads to a significant improvement in the suspension durability. On the other hand, neutralization of the surface charge resulting from the binding of macromolecules or the formation of polymer bridges between the adjacent particles may result in the formation of larger aggregates and then their sedimentation. This is a particularly desirable phenomenon in the case of the treatment of water and sewage containing suspended solids [3,4,5].

Polyamino acids are a group of synthetic polymers whose macromolecules are composed of only one type of unit connected by an amide bond (unlike proteins containing many different peptides in their structure). Taking into account the variety of chemical properties of amino acid molecules (e.g., the ionic nature of functional groups, hydrophobicity, polarity and thermal stability), it is possible to obtain macromolecules characterized by specific features and an appropriate chain structure. Although these compounds are obtained mainly by polymerization of appropriate substrates, some of them, such as poly(L-lysine) or poly(L-glutamic acid), occur in the natural environment, including in the capsules of many bacteria strains, where they prevent excessive cell dehydration. Due to the use of monomers in the form of amino acid molecules in the synthesis process, the obtained macromolecular substances have several properties important for medicine, pharmacy and environmental protection. First of all, the macromolecules are completely biodegradable. Secondly, polyamino acids are biocompatible with the human body; they are also completely non-toxic and do not cause an immune response. Additionally, both the ability to control the synthesized polymer chain structure and the use of amino acid derivatives as monomers contribute to the formation of substances with precisely defined properties. This structure diversity applies especially to polymers composed of three peptides: glutamic acid, aspartic acid and lysine. Side chain modification allows obtaining polymers with a wide range of properties—from hydrophilic compounds with a linear structure and ionic or non-ionic character (the macromolecules in solution assume the random coil conformation), to hydrophobic substances that dissolve only in organic, non-polar solvents and whose macromolecules can form domains structurally similar to liquid crystals. Moreover, due to their chemical and structural similarity to proteins, polyamino acids are often used as simple models for more complex naturally occurring biopolymers [6,7,8]. The variety of biological functions and interesting chemical properties contribute to the constant increase in interest in this polymer group, both in the area of basic research and in the case of their potential applications [9,10,11,12,13].

The interaction between simple polymers and the surface of the corresponding metal oxide have been extensively studied [14,15,16]. The increase in ecological awareness, as well as the search for new materials, has led to greater interest in copolymers—substances having two (or more) types of segments in a macromolecule connected by a permanent bond. Importantly, these compounds exhibit significantly different properties compared to the corresponding homopolymers. Due to the presence of fragments characterized by the different ionic nature or polarity in the chain, the adsorption layers created using block copolymers are characterized by a specific structure [17,18,19].

In the presented paper, the block copolymers of ionic polyamino acids with poly(ethylene glycol) (PEG) were used. The latter belongs to the non-ionic macromolecular compound group obtained by chemical synthesis and is widely used in various industry branches. PEG is characterized by good water solubility, non-toxicity and biocompatibility with the human body. It is possible to chemically bind the PEG chains with drug molecules, peptides and proteins, as well as solids nanoparticles (usually metal oxides), micelles or liposomes. It should be emphasized that block copolymers containing a non-ionic PEG domain are a group of compounds with high application potential used in many fields of medicine, pharmacy and industry [20,21,22,23,24,25,26].

The chromium(III) oxide is used as a stable green pigment in the construction and ceramics industries, a heterogeneous catalyst and a coating providing thermal and mechanical protection. Due to the potential environmental threats related to the Cr_2_O_3_ presence in industrial sewage, it is necessary to develop an effective method of disposal. One of the most promising may be the use of polymer adsorption to destabilize the resulting suspensions [27].

The aim of this work focused on determining the influence of various factors on the polymer adsorption layer structure. The first goal was to characterize the conformation of adsorbed macromolecules depending on the polyamino acid functional group type (constituting thereby a reference system for further analysis). Due to the opposite ionic nature of the polypeptide block, measurements were carried out at different pH values of the solution (equal to 3, 4, 7.6 and 10). The next stage was to determine the influence of the block copolymer structure on its binding mechanism under specific pH conditions. For this purpose, polymeric substances with similar molecular weights but different contents of individual segments of ionic polyamino acid and PEG (diblock and selected triblock copolymer) were used. It is worth noting that maintaining a similar molecular weight of the entire compound enables comparison of the collected adsorption data. The last analyzed parameter was the influence of the non-ionic fragment length present in the chain structure of diblock compounds on the polymer binding to the solid surface.

## 2. Results and Discussion

### 2.1. The Structure of the Polymer Adsorption Layer in the Cr_2_O_3_/Ionic Polyamino Acid System

The dissociation degree (denoted as α) values of the ionic polyamino acids calculated based on the Gran method are presented in Table 1. As can be seen, the selected polymers exhibit the opposite chemical nature, and the influence of the solution pH on the polymer chain structure should be considered. Additionally, the pH impact on the changes in the charge accumulated on the Cr_2_O_3_ surface should be taken into account. The sorbent surface has a decisive influence on the polymer chain arrangement, being one of the crucial factors determining the polymer binging. 

**Figure 1:**
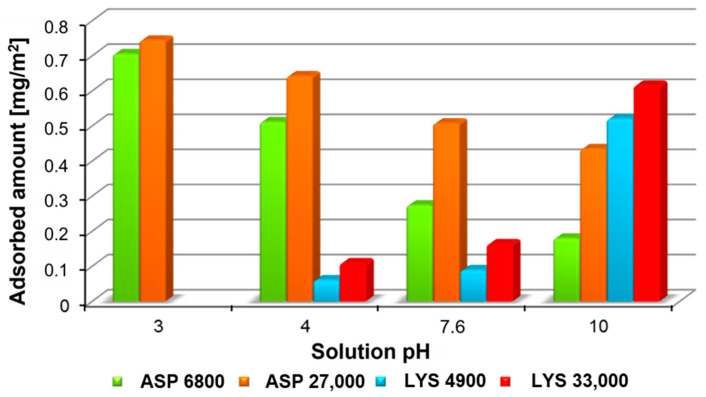
Comparison of the ionic homopolymers adsorption amounts at different solution pH values (polymer concentration = 100 mg/L). The LYS adsorption was not observed at pH = 3.

The dependence of the analyzed macromolecular compounds’ molecular weight and the solution pH on the polymer adsorption amount on the colloidal Cr_2_O_3_ surface is shown in Figure 1. The anionic polymer adsorption reaches a maximum in the low pH region. The ASP binding mechanism under these conditions can be explained based on electrostatic interactions and hydrogen bond formation. The low ASP dissociation degree favors the formation of a more folded structure of adsorbing macromolecules. In an alkaline environment, the ASP affinity for the adsorbent decreases rapidly due to the presence of electrostatic repulsion forces. Moreover, the developed conformation of the chains located in the solid surface layer impedes access to the adsorbent active sites for the subsequent polymer macromolecules. The opposite situation occurs in solutions containing LYS. This difference can be explained by the different ionic nature of poly(L-lysine), which in turn determines the manner of the macromolecule’s interaction with the Cr_2_O_3_ surface. In this case, the polymer adsorption reaches the maximum at pH 10, where the electrostatic attraction between the suspension constituents is strongest. The lowest adsorption was obtained at pH 4 (no adsorption was observed at pH 3). It is a consequence of blocking the adjacent adsorbent active sites by chains characterized by a stretched conformation. Under these conditions, the hydrogen bridges are primarily responsible for the polymer binding process. Moreover, due to the larger number of –CH_2_ groups present in the side chain fragments to which the polymer functional groups are connected (compared to the ASP structure), the poly(L-lysine) adsorption mechanism should additionally take into account the hydrophobic interaction occurrence. The analysis of the data in Figure 1 reveals that the molecular weight strongly affects the polymer adsorption. It follows from the polymer adsorption layer structure. In general, the more functional groups capable of interacting with the solid surface in a macromolecule chain, the richer the resulting structure is in the polymer segments forming so-called loops and tails. 

**Figure 2:**
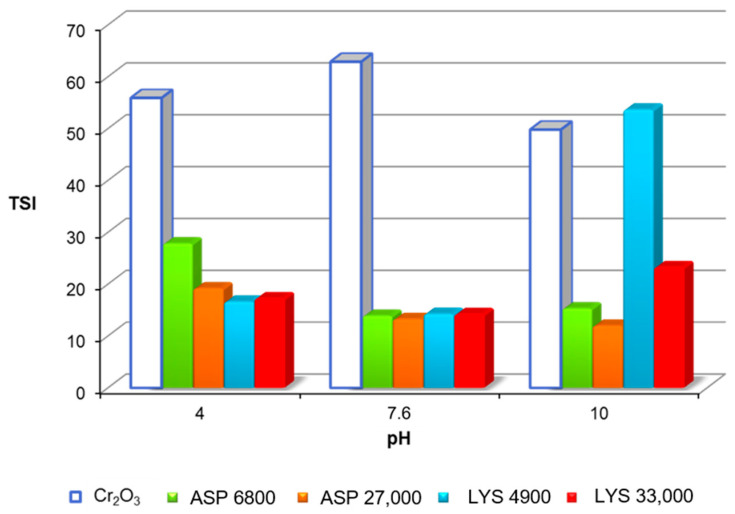
TSI parameter values calculated for Cr_2_O_3_ suspension without and in the presence of the tested homopolymers (c = 100 mg/L).

In order to precisely determine the durability of the tested systems, turbidimetric measurements were conducted, including the calculation of the TSI parameter, shown in Figure 2. The TSI parameter values indicate that the Cr_2_O_3_ suspensions without the polymer addition are quite unstable. 

At pH 4, the Cr_2_O_3_ particles contain mainly positively charged surface groups; however, under these conditions, there can exist a certain amount of ≡CrO^−^ groups. It is clearly understood that attraction forces between oppositely ionized groups lead to the particle’s aggregation and therefore system destabilization. At pH 7.6, the system exhibits minimum stability (TSI = 62.91). Under these conditions, the overall surface charge is equal to zero which means that there is the same number of positively and negatively charged groups. Increasing the contribution of attraction between differently charged adsorbent surface groups is responsible for the stability reduction (this is also confirmed by the zeta potential value). The higher the absolute value of the zeta potential, the greater the probability that the studied suspension will be stable. A small value of the ζ potential (from +5 to −5 mV) indicates a tendency for system destabilization. The colloidal suspensions exhibit the smallest stability at the isoelectric point, where the total charge of the diffusion layer around the particles is equal to zero. Merging the solid particles into larger aggregates leads to their faster sedimentation, resulting in lower stability of the system. Relatively, the highest TSI parameter value was reached at pH 10 (TSI = 49.82). This can be driven by electrostatic repulsion between the negative surface groups.

The presence of the macromolecular compounds significantly changes the durability of the Cr_2_O_3_ suspension (Figure 2). Both the addition of anionic poly(L-aspartic acid) and cationic poly(L-lysine) increase the stability of the system compared to the suspension in the absence of the polymer (except for LYS 4900 at pH 10). The high adsorption of ASP, which is the effect of electrostatic attraction between the positively charged adsorbent surface and the dissociated carboxyl groups present in the anionic polymer chain, is responsible for improving the stability of the Cr_2_O_3_ suspension at pH 4. Under these conditions, ASP chains exist in the most coiled form, which leads to significant surface coverage with the polymer macromolecules. The formation of a compact adsorption film promotes steric stabilization, where mutual repulsive forces between polymer-coated particles hinder the formation of larger aggregates. Moreover, the flocculation of colloidal particles is prevented by the presence of charge in the polyamino acid adsorption layer. At pH 7.6, the ASP adsorption process occurs mainly due to the hydrogen bond formation. Additionally, a high degree of polymer dissociation causes an increase in repulsive forces between the adsorbed chains. Under these conditions, a loosely packed adsorption layer is formed in which numerous negatively charged functional groups are directed towards the bulk phase, ensuring electrostatic stabilization of the analyzed system due to electrostatic repulsion between the adsorption layers of colloidal particles. The solution pH increase causes further development of polymer chains located on the Cr_2_O_3_ surface and the formation of a spatial structure rich in long loops and tails. The repulsion between ASP coated Cr_2_O_3_ particles is additionally enhanced by the presence of numerous unadsorbed macromolecules in the solution (so-called depletion stabilization).

For the ASP polymer, the influence of the molecular weight on the polymer stabilization properties (compared to the chromium(III) oxide suspension) is visible. The addition of a lower-mass homopolymer results in a significant increase in the stability of the suspension. The application of a polyamino acid with a larger molecular weight follows from a further increase in the Cr_2_O_3_ suspension durability. As a result, a more closely packed adsorption layer is created, which increases the steric repulsion contribution between the adjacent colloid particles.

The presence of poly(L-lysine) improves the Cr_2_O_3_ suspension stability (except for the system containing LYS 4900 at pH 10). At a pH of 4, the cationic polyamino acid undergoes the weakest adsorption, mainly due to the hydrogen bond formation and hydrophobic interactions. At the same time, the fully protonated amino groups cause the formation of a spatially developed adsorption layer with a positive charge located on the Cr_2_O_3_ surface. As a consequence, the mutual repulsion of the charged colloid particles coated with LYS is the main reason for the increase in suspension durability (compared to the systems without the mentioned polymer). In addition to the electrosteric effect, stabilization related to the presence of numerous unadsorbed LYS macromolecules in the solution plays an important role.

Visible changes in the Cr_2_O_3_ suspension stability in the presence of LYS were observed at pH 10. Concerning the chromium(III) oxide suspension without polymer, the polyamino acid with a lower mass causes a slight reduction in the system’s stability (TSI = 53.64), while the addition of LYS 33,000 improves its durability (TSI = 23.24). In the alkaline environment, the LYS adsorption increases due to the electrostatic attraction forces. Simultaneously, a decrease in the polymer dissociation degree allows the formation of a more compact adsorption layer and promotes the formation of hydrogen bonds between the Cr_2_O_3_ surface and poly(L-lysine) chains. A reduction in the number of positively charged functional groups in the diffusion layer leads to a decrease in electrostatic repulsion forces. The formation of polymer bridges between adsorption layers of various colloidal particles (bridge flocculation) and the increase in the strength of hydrophobic interactions between polymer layers adsorbed on adjacent solid particles are responsible for the destabilization of the analyzed suspension in the presence of LYS 4900. The addition of a polymer with a larger molecular weight provides to obtain the more developed, spatial polymer conformation. The increase in the adsorption layer thickness contributes to the suspension stability improvement due to the presence of steric effects.

### 2.2. Binding of Diblock and Copolymers with Poly(ethylene glycol) on the Chromium(III) Oxide Surface

Studies on the binding mechanism of block copolymers of polyamino acids with poly(ethylene glycol) included the determination of the adsorption dependencies in the systems containing different copolymers characterized by similar molecular weights. To precisely define the forces present in the tested systems, the results obtained for appropriate homopolymers and macromolecular compounds with a block structure were compared. Moreover, the analysis of data obtained from suspension stability measurements allowed for the determination of the mechanism of stabilization/destabilization of aqueous Cr_2_O_3_ suspensions in the presence of the selected copolymers.

**Figure 3:**
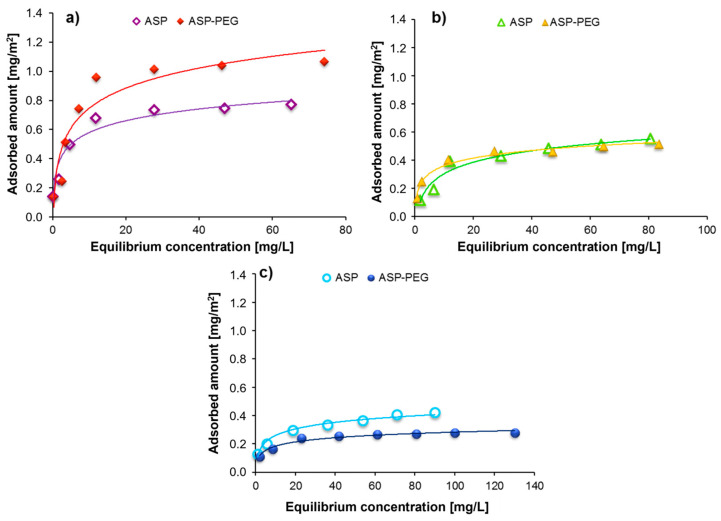
Comparison of the adsorption isotherms obtained for the ASP homopolymer (Mw = 27,000) and the ASP–PEG 27-1 copolymer (containing the polyamino chain of Mw = 27,000 Da) in the solutions of different pH: (**a**) pH 3, (**b**) pH 7.6, (**c**) pH 10.

Comparison of the adsorption isotherms obtained for the ASP homo- and copolymers for the different solution pH are presented in Figure 3. At pH 3, the binding of anionic polyamino acid at the Cr_2_O_3_–solution interface is based mainly on the electrostatic interactions between the positively charged adsorbent surface groups and the dissociated carboxyl moieties of ASP. Higher adsorption of the ASP–PEG 27-1 copolymer is possible due to the formation of a mixed polymer film—both blocks are directly bonded on the Cr_2_O_3_ surface. Furthermore, the non-ionic PEG segments can form hydrogen bonds with the undissociated carboxyl groups of adjacent ASP chains, which further stabilizes the overall structure. Under the same conditions, poly(L-aspartic acid) can only interact with the solid surface through hydrogen bonds or electrostatic attraction forces. At pH 7.6 (pHpzc of the oxide), a sharp decrease in the adsorption amount of both polymers is observed compared to pH 3. Additionally, the adsorption of the diblock copolymer is gradually reduced compared to ASP. This comes from the solid surface charge changes and the increase in the dissociation degree of the block originating from ASP segments. As a consequence, a reduction in the electrostatic interactions between the ASP block and the Cr_2_O_3_ surface is noted. Moreover, the higher dissociation degree favors the formation of a more extended conformation of the adsorbed polymer chains. Further increasing the solution pH causes a decrease in the polymer’s adsorption amount. At pH 10, the non-ionic fragment of the ASP–PEG 27-1 copolymer macromolecule behaves like a buoy, and due to the lack of affinity for the Cr_2_O_3_ surface, it does not participate in adsorption at the phase boundary. At the same time, such an arrangement of the non-ionic part of the copolymer chain may contribute to the blocking of a larger number of solid active sites, causing a marked decrease in copolymer adsorption in comparison to ASP.

**Figure 4:**
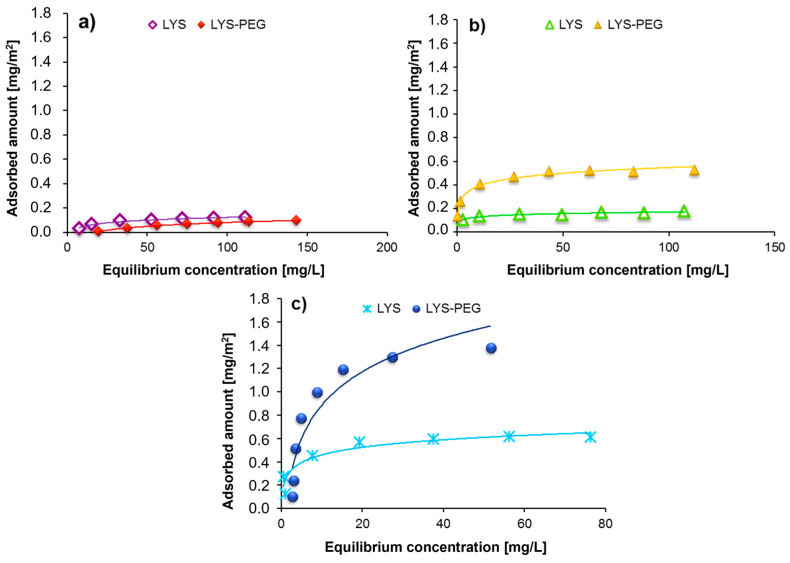
Comparison of the adsorption isotherms obtained for the poly(L-lysine) homopolymer (Mw = 33,000) and the LYS–PEG 33-1 copolymer (containing the polyamino chain of Mw = 33,000 Da) in the solutions of different pH: (**a**) pH 4, (**b**) pH 7.6, (**c**) pH 10.

The analysis of the adsorption data obtained for poly(L-lysine) and LYS–PEG copolymer (Figure 4) clearly shows that in both cases most macromolecules are bound to the adsorbent surface at pH 10. It should be emphasized that despite the presence of a PEG block in the polymer chain, the copolymer adsorption at pH 3 is not observed. The order of the curves is also important—the introduction of a non-ionic fragment into the macromolecule structure contributes to the increase in the adsorption of LYS–PEG 33-1 compared to the homopolymer itself in an alkaline or neutral environment. The reverse order of adsorption isotherms was observed at pH 4—the LYS homopolymer is strongly bounded on the Cr_2_O_3_ particles’ surface. Under these conditions, the poly(ethylene glycol) segments act as an “anchor” by forming hydrogen bonds with numerous positively charged solid surface groups. The decrease in the number of LYS–PEG 33-1 macromolecules adsorbed on the Cr_2_O_3_ surface is the result of “obscuring” the adsorbent active sites by a much longer LYS block, characterized by a spatially developed conformation. The increase in the adsorption of LYS–PEG 33-1 in comparison to a homopolymer of very similar mass at pH 7.6 and 10 can be explained by the impact of the PEG fragment presence in the copolymer structure. In the alkaline environment, the non-ionic block does not adsorb on the Cr_2_O_3_ surface, but it can interact with poly(L-lysine) segments of other macromolecules, forming hydrogen bonds. Such a shielding of charges coming from neighboring adsorbed chains leads to an increase in the adsorption amount of the copolymer compared to poly(L-lysine). Under these conditions, the copolymer adsorption provides the formation of the polymer film, in which both diblock copolymer structural units are directly bonded to the adsorbent surface.

A comparison of the TSI values in the presence of ASP and ASP–PEG (Table 2) indicates that the structure of the polymer macromolecules has a crucial influence on the adsorption layer structure and, therefore, on the durability of the analyzed systems. At pH 3, the adsorption of both concerned substances causes deterioration of the suspension stability compared to the system without the polymer. The reason for this situation is the saturation of the adsorbent surface charge by the polymer chains located at the solid–liquid interface. Moreover, the low degree of dissociation of carboxyl groups and the presence of the PEG block favor the formation of hydrogen bridges between adjacent solid particles. At the point of zero charge (pH = 7.6), an improvement in the stability of systems containing anionic polymers is observed compared to Cr_2_O_3_ particles dispersed in a background electrolyte solution. At the same time, the increase in durability is varied and depends on the structure of the compound used, even though the adsorption rate of both polymeric substances is very similar (TSI equal to 13.22 and 33.11 for ASP and ASP–PEG, respectively). It is worth noting that under these conditions, the extension of long chains towards the bulk phase contributes to a more effective repulsion of polymer-coated particles. In the system containing ASP–PEG, the presence of numerous hydrogen bridges formed between the segments of both blocks adsorbed on the surface of neighboring colloidal particles is the main reason for the lower stability of the suspension compared to the one containing ASP. In an alkaline environment, the non-ionic chain fragment has practically no affinity for the surface and is a buoy directed towards the solution [28,29,30]. Therefore, it can create hydrophobic interactions or form hydrogen bonds with segments of both blocks bound to the surface of other solid particles. Importantly, a decrease in the copolymer adsorption amount, due to blocking the adsorbent active sites, results from a smaller number of the negatively charged polyamino acid functional groups introduced into the diffusion layer. In the ASP binding case, the presence of the dissociated carboxylic groups in the homopolymer macromolecules in the loops and tails form promotes greater stability of the Cr_2_O_3_ suspension due to electrosteric stabilization.

The analysis of the TSI data obtained for the systems containing the cationic polymers (or copolymers; Table 3) shows that at pH 4 the stability parameter reaches higher values in the LYS–PEG/Cr_2_O_3_ system compared to the sample containing LYS (it should be noted that under these conditions the lower adsorption of the copolymer is observed). The block copolymer macromolecules with a highly developed chain conformation adsorbed on the surface hinder access to the remaining solid active sites. Hence, the anchor in the form of a short PEG fragment plays a key role in the mechanism of the copolymer binding to the mineral oxide surface. Despite the complete ionization of the amino groups located mainly in the solid diffusion layer, hydrogen bonds may be formed between the segments of different blocks, which explains the higher TSI value compared to the LYS/Cr_2_O_3_ system. In the case of a suspension containing poly(L-lysine), the repulsive forces between the numerous loops and tails located in the adsorption layer provide suspension stabilization. Under pHpzc conditions and at pH 10, both cationic polymers contribute to increasing the stability of aqueous Cr_2_O_3_ suspensions. It should also be noted that this improvement is much less visible after the use of a block copolymer (at pH 10, the difference in TSI values for LYS–PEG/Cr_2_O_3_ and Cr_2_O_3_ systems is only 5 units). Above the point of zero charge, the adsorption of both compounds increases rapidly, mainly due to electrostatic attraction. At the same time, the degree of ionization of the amino groups in the poly(L-lysine) segments gradually decreases, therefore the adsorbed chains adopt a more folded conformation. In the case of LYS, a larger number of positively charged loops and tails in the diffusion film results in an increase in the repulsive forces between neighboring particles. The slight deterioration in stability observed in the Cr_2_O_3_/LYS system at pH 10 can be explained by the appearance of single polymer bridges.

### 2.3. The Influence of Poly(L-aspartic Acid) Chain Length on the Copolymer Adsorption Mechanism at the Cr_2_O_3_ Surface

**Figure 5:**
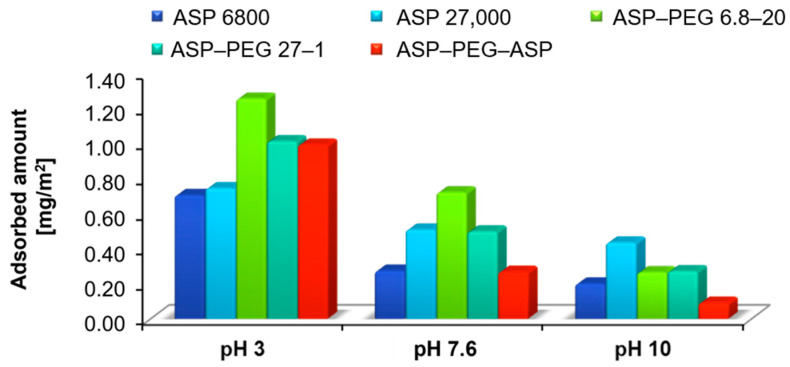
Comparison of the adsorption amounts obtained for poly(L-aspartic acid) with different Mw and its block copolymers (c = 100 mg/L) for three solution pH values.

To determine the most probable structure of the adsorption layer, the polymers with different ASP chain lengths were used: the anionic homopolymer—poly(L-aspartic acid) and its block copolymers with PEG (diblock ASP–PEG 6.8-20, ASP–PEG 27-1 and triblock ASP–PEG–ASP; Table 1). The comparison of the adsorption amounts in the systems containing the above-mentioned polymers is shown in Figure 5.

As can be seen from Figure 5, the adsorption maximum for all substances containing the anionic polyamino acid is reached at pH 3. Under these conditions, the binding process is driven by the attractive forces between the partially dissociated functional groups of the poly(L-aspartic acid) segments and the Cr_2_O_3_ surface. Among the analyzed block copolymers, the highest adsorption was observed in the system containing ASP–PEG 6.8-20, while similar adsorption rates were obtained for ASP–PEG 27-1 and ASP–PEG–ASP triblock copolymer. To explain the presented relationships, the results obtained for homopolymers and block polymers with a similar molecular weight of the polyamino acid chain should be compared.

In the case of the compounds containing the ASP fragment of the same length (ASP 6800 and ASP–PEG 6.8-20), the diblock copolymer exhibits almost twice as much adsorption compared to poly(L-aspartic acid). It can be explained based on the PEG’s ability to form hydrogen bonds with the Cr_2_O_3_ surface. Moreover, in this system, a mixed layer may be formed, in which both blocks are directly bonded at the phase boundary. Additionally, the numerous non-ionic polymer segments can screen charges belonging to adjacent ASP chains. The analysis of data obtained for ASP 27,000 and ASP–PEG 27-1 showed that the introduction of a small PEG fragment into the macromolecule structure improves the adsorption properties of the block copolymer. However, due to the lower molecular weight of the PEG chain (concerning ASP–PEG 6.8-20), the created adsorption polymer layer is characterized by a looser packing of polymer coils. A short PEG fragment is unable to ensure adequate isolation of the charges present in the polyamino acid segments.

At higher pH (7.6 and 10), the adsorption of all tested compounds decreases significantly. The main reason is the electrostatic repulsion appearance between the suspension components. Moreover, the extended macromolecules’ conformation impedes the hydrogen bond formation. It should also be noted that at pH 10 the PEG block loses its affinity to the mineral oxide surface. As a result, the adsorption layer containing the non-ionic loops, constituting a steric obstacle, is formed. In the alkaline environment, the ASP with a molecular weight of 27,000 Da exhibits higher adsorption in the homopolymers group; however, the number of bound macromolecules of both diblock copolymers is similar. The smallest number of chains located at the Cr_2_O_3_–solution interface was recorded in the system containing symmetric triblock copolymer ASP–PEG–ASP. Similarly to pH 3, the number of adsorbed ASP–PEG 6.8-20 macromolecules is higher compared to ASP 6800 due to the possibility of hydrogen bond formation and the ability to screen the charge of the polyamino acid chains by the long PEG block. In the case of the pair of compounds ASP 27,000 and ASP–PEG 27-1, a marked decrease in the diblock copolymer adsorption is observed. The increase in the number of the dissociated carboxyl groups in the ASP–PEG 27-1 structure contributes to an increase in the share of repulsive forces. In this situation, a short fragment of the non-ionic polymer ineffectively eliminates the electrostatic repulsion between adjacent adsorbed ASP chains. A similar binding mechanism can be proposed for the Cr_2_O_3_/ASP–PEG–ASP system, where two long ASP chains are connected via short non-ionic PEG.

## 3. Materials and Methods

**Figure 6:**
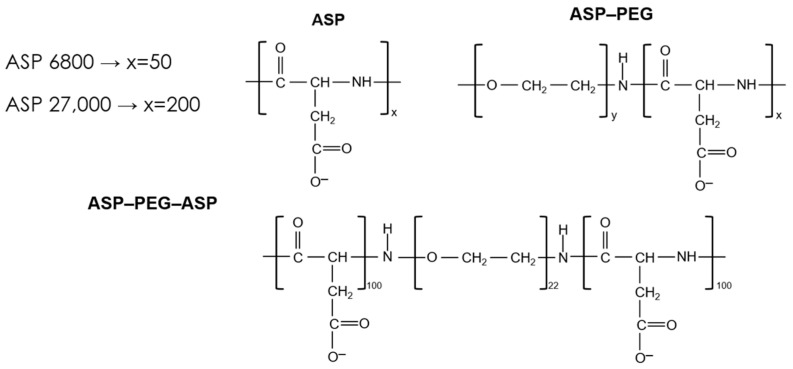
Structure of the polymers containing anionic polyamino acid–poly(L-aspartic acid) along with determining the number of individual segments (in the case of block copolymers).

Ionic polyamino acids and their block copolymers with poly(ethylene glycol) (PEG) purchased from Alamanda Polymers Inc. (Huntsville, AL, USA) were used in the research. The studied macromolecular substances were characterized by a low polydispersity coefficient ranging from 1.02 to 1.2. These compounds can be divided into three groups. The first contains the simple homopolymers: anionic poly(L-aspartic acid), sodium salt (denoted as ASP) and cationic poly(L-lysine) hydrochloride (denoted as LYS). The average molecular weights (M_w_) of the mentioned polymers determined by the manufacturer were 6800 and 27.000 for ASP and 4900 and 33,000 for LYS, respectively (Figure 6). Another group of macromolecular compounds were diblock copolymers (characterized by different lengths of polyamino acid and PEG blocks) and symmetrical triblock copolymers (Table 4). The abbreviation ASP–PEG refers to a block copolymer composed of poly(L-aspartic acid) and poly(ethylene glycol) chains. Analogously, LYS–PEG denotes a block copolymer composed of poly(L-lysine) and poly(ethylene glycol) units. In the case of the symmetric triblock copolymer, ASP–PEG–ASP, the short non-ionic block consisting of poly(ethylene glycol) was connected to two long polyamino acid chains. The dissociation constant (pKa) values measured using the potentiometric titration method were 3.73 and 10.55 for ASP and LYS, respectively [31].

**Figure 7:**
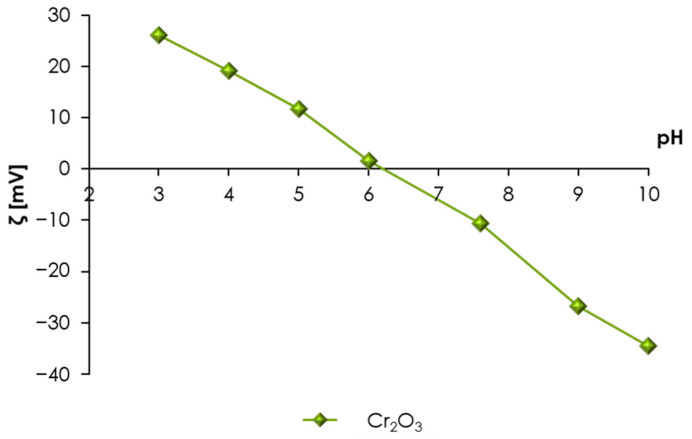
The zeta potential obtained for the Cr_2_O_3_ suspension in the presence of a background electrolyte.

Chromium(III) oxide (Cr_2_O_3_) produced by Polskie Odczynniki Chemiczne (Gliwice, Poland) was used as an adsorbent. The specific surface area of the mentioned solid determined by the BET method (analysis of nitrogen adsorption–desorption isotherms; Micromeritics ASAP 2405 analyzer, Norcross, GA, USA) was found to be 7.12 m^2^/g. The following properties were determined to obtain the sorbent surface characteristics: the point of zero charge (pHpzc) of Cr_2_O_3_ was 7.6 (obtained from the potentiometric titration), and its isoelectric point (pHiep) was about 6 (zeta potential measurements; Zetasizer 3000, Malvern Instruments, Almelo, The Netherlands). The zeta potential curve in different pH values obtained for Cr_2_O_3_ is shown in Figure 7.

The average Cr_2_O_3_ particle size measured using dynamic light scattering was equal to 265 nm with the polydispersity index value below 0.25 [31,32].

Adsorption measurements were carried out in the following way. The tested polymer solutions were prepared from a stock solution with a concentration of 1000 mg/L. Then, the appropriate solid mass was added to the Erlenmeyer flask and the desired pH value was set using the HCl or NaOH solutions. The prepared samples were placed in a water shaker (120 rpm, 25 °C) for 22–24 h to achieve adsorption equilibrium. In the next step, the solid was centrifuged twice for 10 min at 4000 rpm (MPW-223e centrifuge, MPW Med. Instruments, Warsaw, Poland) and the clear solution was used to determine the polymer concentration spectrophotometrically (Cary 100, Varian, Agilent Technology, Santa Clara, CA, USA). The absorbance of the solutions was measured at 210 nm (the correction for the solution pH effect, especially in the alkaline solutions, was taken into account) [33]. All measurements were carried out in quartz cuvettes, using redistilled water as the reference solution. The range of the tested concentrations was from 10 to 200 mg/L. The polymer concentration after the adsorption process was calculated using the calibration curves of the analyzed compounds. 

To investigate the polymer adsorption influence on the colloidal systems stability, turbidimetric measurements were performed using the Turbiscan LabExpert coupled with the TLab Cooler (both purchased from Microtrac Inc., York, PA, USA). The use of computer software (TLab EXPERT 1.13) allowed the calculation of the TSI (TSI, Turbiscan Stability Index, is a specific parameter intended for comparison and characterization of the physical stability of various formulations). This parameter is extremely helpful in estimating the stability of the tested colloidal systems. TSI values range from 0 to 100, where the lower the value, the greater the stability the sample exhibits. The solid suspension was prepared as follows: the adsorbent was added to the background electrolyte solution. Then, the desired pH was set following the polymer addition. Afterwards, the sample was intensively shaken and inserted into a thermostated measuring device. 

All measurements were performed in the pH range 3–10 at room temperature (≈25 °C), and the polymer concentration was 100 mg/L. NaCl of concentration 0.01 mole/dm^3^ was used as a supported electrolyte.

## 4. Conclusions

The homopolymer adsorption depends on the pH of the measuring system. For an anionic polymer, this process takes place most effectively in an acidic environment, while the fewest ASP macromolecules are bound to the Cr_2_O_3_ surface at pH = 10. In addition, the introduction of the ASP segments on the Cr_2_O_3_ surface significantly improves the stability of the Cr_2_O_3_ suspension due to the increase in the repulsion forces between negatively charged polymer chains. The opposite behavior was exhibited by cationic poly(L-lysine), which was most strongly bound in alkaline solutions. In this case, the spatially developed conformation of macromolecules contributes to increasing the stability of the chromium(III) oxide suspensions at pH 4 and 7.6. In an alkaline environment, the neutralization of the Cr_2_O_3_ particle’s surface charge leads to a reduction in the stability of the colloidal system.

The polymer adsorption film structure formed at the metal oxide–block copolymer solution interface depends on the following factors. First of all, the amount of adsorbed chains is influenced by the type of ionic functional groups present in the polymer macromolecules. Another parameter that determines the arrangement of the chains is the solution pH, which affects the interactions between the suspension components. However, the ratio of the ionic/non-ionic block length is equally important. The presence of a PEG fragment in the main chain of diblock and triblock copolymers changes their binding mechanism compared to the corresponding homopolymers. This can be related to the formation of a mixed layer composed of both blocks directly bonded to the adsorbent surface or shielding of charges present in the polyamino acid segments.

## Figures and Tables

**Table 1:** Values of the poly(L-lysine) and poly(L-aspartic acid) dissociation degree (α) and types of Cr_2_O_3_ surface groups in the analyzed pH range.

pH	α ASP [%]	α LYS [%]	Surface Groups
3	15.7	100.0	Mainly ≡CrOH_2_^+^
4	65.1	100.0	Mainly ≡CrOH_2_^+^
7.6	99.9	99.9	Equal amounts of ≡CrOH_2_^+^ and ≡CrO^−^
10	100.0	78.0	Mainly ≡CrO^−^

**Table 2:** TSI values comparison in the systems without and after the addition of poly(L-aspartic acid) containing macromolecular compounds (c = 100 mg/L).

	TSI Values		
pH	Cr_2_O_3_	Cr_2_O_3_/ASP	Cr_2_O_3_/ASP–PEG
3	12.76	48.71	47.19
7.6	62.91	13.22	33.11
10	49.82	11.94	35.55

**Table 3:** TSI values comparison in the systems without and after the addition of poly(L-lysine) containing macromolecular compounds (c = 100 mg/L).

	TSI Values		
pH	Cr_2_O_3_	Cr_2_O_3_/LYS	Cr_2_O_3_/LYS–PEG
4	12.76	17.24	26.21
7.6	62.91	14.18	35.21
10	49.82	23.24	44.85

**Table 4:** The molecular weights of the individual block building the diblock and triblock copolymers.

Polymer Name	M_w_ of the Polyamino Acid Ionic Block [Da]	M_w_ of the PEG Block [Da]
ASP–PEG 6,8-20	6800	20,000
ASP–PEG 27-1	27,000	1000
ASP–PEG–ASP	14,000	1000
LYS–PEG 33-1	33,000	1000

## Data Availability

Data are contained within the article.
